# PI3K/Akt/HIF-1α signaling pathway mediates HPV-16 oncoprotein-induced expression of EMT-related transcription factors in non-small cell lung cancer cells: Erratum

**DOI:** 10.7150/jca.87270

**Published:** 2023-07-06

**Authors:** Jinhua Liu, Bingyu Huang, Zihan Xiu, Zhiyuan Zhou, Jiao Liu, Xiangyong Li, Xudong Tang

**Affiliations:** 1 Institute of Biochemistry and Molecular Biology, Guangdong Medical University, Zhanjiang 524023, P.R. China; 2 Collaborative innovation center for antitumor active substance research and development, Guangdong Medical University, Zhanjiang 524023, P.R. China; 3 Dongguan Key Laboratory of Medical Bioactive Molecular Developmental and Translational Research, Guangdong Medical University, Dongguan 523808, P.R. China; 4 Guangdong Provincial Key Laboratory of Medical Molecular Diagnostics, Guangdong Medical University, Dongguan 523808, P.R. China.

In the original version of our article, there was an inadvertent error in Figure 1. Specifically, the result of β-actin expression of NCI-H460 cells in the right of Figure 1B was wrongly pasted. The correct Figure 1 is provided below. This correction will not affect the results and conclusions. The authors apologize for any inconvenience the error may have caused.

## Figures and Tables

**Figure 1:**
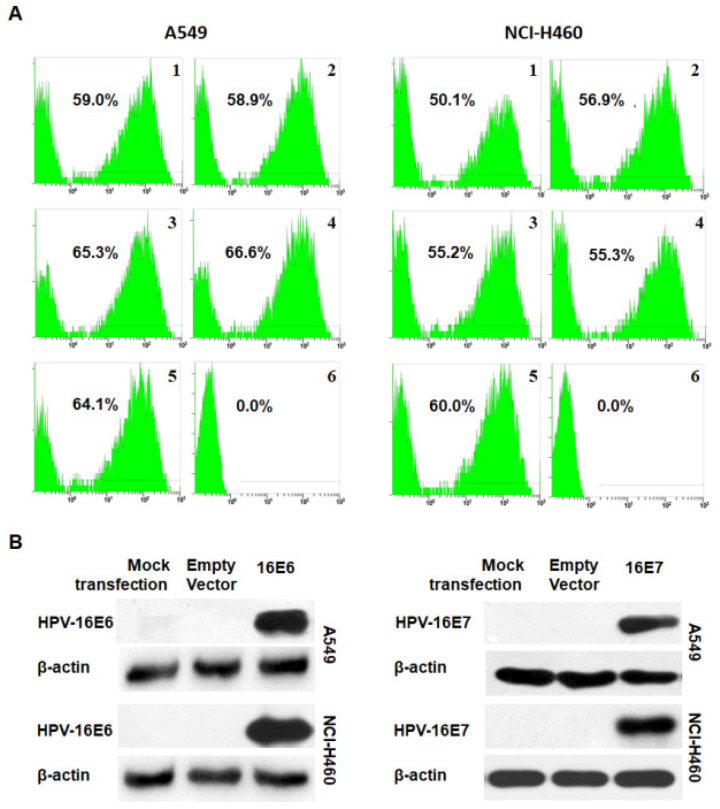
Analysis of transfection efficiency. (A) The transfection efficiency was analyzed by flow cytometry in transfected A549 (left) and NCI-H460 (right) cells. 1. HPV-16E6-transfected cells; 2. HPV-16E6 mutant-transfected cells; 3. HPV-16E7-transfected cells; 4. HPV-16E7 mutant-transfected cells; 5. Empty vector-transfected control cells; 6. Mock-transfection control cells. (B) The expression of HPV-16E6 (left) and HPV-16E7 (right) oncoproteins was determined by Western blot analysis.

